# Comparison of conventional impression making and intraoral scanning for the study of unilateral cleft lip and palate

**DOI:** 10.1111/cga.12499

**Published:** 2022-11-24

**Authors:** Tomoyo Okazaki, Hitoshi Kawanabe, Kazunori Fukui

**Affiliations:** ^1^ Division of Orthodontics and Dentofacial Orthopedics, Department of Oral Growth and Development Ohu University School of Dentistry Koriyama City Fukushima Japan

**Keywords:** alveolar, cleft lip and palate, nasoalveolar molding, three‐dimensional analysis, unilateral

## Abstract

Patients with cleft lip and palate (CLP) encounter various problems, including disorders related to feeding, esthetics, and pronunciation. We compared two impression methods, conventional impression making and intraoral scanning, to study unilateral cleft lip and palate (UCLP). Patients with UCLP (*n* = 7) were selected, and palatal impressions were taken by two steps: (1) impressions were obtained using an addition silicone rubber impression material, and a plaster model was prepared and (2) optical impressions were obtained using a desktop three‐dimensional (3D) scanner and stereolithography (STL). Data were generated by two impression system combinations through STL. The results were analyzed using the Kruskal–Wallis or Mann–Whitney *U* test. There were no significant differences in the dimensions of the models between both groups. The measured depth of the alveolar cleft defects was deeper in the plaster model group (STL) than in the intraoral scanner group (STL). Digital models may prevent the risk of aspiration and respiratory disorders by using impression materials for preoperative jaw treatment of newborns and infants. We compared the results of both impression methods in the same patient and found that a shift to the 3D printer model is a safe alternative for preoperative jaw correction, as evidenced from the amount of tissue displaced due to the pressure applied during impression taking. In the future, we would like to conduct clinical research with a larger sample size of CLP patients to further corroborate these findings.

## 1 INTRODUCTION

Presurgical infant orthopedics was introduced by McNeil in 1950, and presurgical orthopedic treatment for patients with cleft lip and palate (CLP) has evolved significantly since.^1^ Patients with CLP have a wide range of problems, including disorders related to feeding, esthetics, and pronunciation.^2^ Of these, the most common problem observed immediately after birth is lactation disorder; hence, successful recovery of feeding function is vital for a child's growth. Primary alveolar surgical repair is performed at an early stage of life for patients with unilateral cleft lip and palate (UCLP) and often before preoperative orthopedic treatment (PSOT) using an acrylic intraoral passive molded plate. Without appropriate early management, UCLP leads to the formation of severe nasal deformities and small alveolar segments.^3^ Therefore, orthopedic procedures are performed in the early stages of life. UCLP usually begins at the lip and proceeds toward the palate; this not only impairs jawbone growth and feeding of the child but also results in considerable psychological stress for the lactating mother.^4^ Grayson et al. reported that passive acrylic plate nasoalveolar molding (NAM), which mimics the form of a normal palate with the use of adhesive paste or slim adhesive tapes fixed from the hook to the cheeks, is a form of orthopedic treatment that encourages breastfeeding and improves psychological stress experienced by breastfeeding mothers.^4^, ^5^, ^6^ Preoperative jaw correction, using a passive acrylic plate mimicking nasolabial and alveolar morphology, is performed shortly after birth with the goals of ameliorating feeding disorders in infants with CLP and correcting morphological abnormalities of the lips, nose, and maxillary alveolar morphology.

However, at any facility, impressions are taken using alginate or silicone impression materials, and treatment is always associated with the risk of accidental ingestion, aspiration, and suffocation of the patient.^7^ We wondered if it would be possible to establish a protocol for PSOT using an intraoral scanner that is safer than conventional impression making.

Optical impressions are taken using an intraoral scanner, which is necessary while making a diagnostic model required for orthodontic treatment and creating a device. However, in Japan, most dental hospitals have not yet used optical impressions except for a few university hospitals. In addition, optical impression taking has been developed for the purpose of obtaining impressions of teeth, and it is difficult to obtain an impression of the toothless jaw of a child with CLP. Hence, this technique has not fully advanced in Japan and abroad. Moreover, preoperative jaw correction by optical impression acquisition for the management of CLP in children has been studied at facilities worldwide; however, no protocol has yet been established. In Japan, 1.43 in 1000 children are born with CLP every year, making it the most common of all congenital diseases.^8^ Therefore, research on this condition is vital and should be advanced.

Fabrication of palatal molds via optical impression taking is more likely to result in favorable preoperative jaw correction than conventional procedures.^2^

Few reports^2^ have been published detailing the use of an intraoral scanner for preoperative jaw correction in children with CLP. In this study, we examined whether combining the use of a three‐dimensional (3D) morphometric model and a digital model using an intraoral scanner could prove useful for safe preoperative jaw correction in children with CLP. Moreover, we aimed to compare the efficacy of this new approach to that of the conventional plaster‐mold model.^9^

## 2 MATERIALS AND METHODS

The patients visited the orthodontic clinic of Ohu University Dental Hospital from December 2018 to August 2020. Preoperative jaw orthodontic treatment was deemed necessary; hence, consent to perform optical impression was obtained from the respective guardians. Impressions were taken in the presence of a physician using an oxygen saturation meter and heart rate monitor. Parents were informed at the time of the first impression about the risk of startup obstruction due to aspiration of the impression material, and impressions were taken only after consent was obtained. Because aspiration of milk due to the vomiting reflex is also a risk factor, we instructed the parents to allow the patients to fast for 4 h before the impression was taken. In this study, seven infants with UCLP (five boys and two girls: 2–6 months old; mean age, 108 days) were included. This study was approved by the ethics committee of the Ohu University School of Dentistry (2018‐12, 2020‐12). The guardians provided written informed consent to obtain optical impressions of the palate of their infants.

### 2.1 Impression‐taking method

**FIGURE 1:**
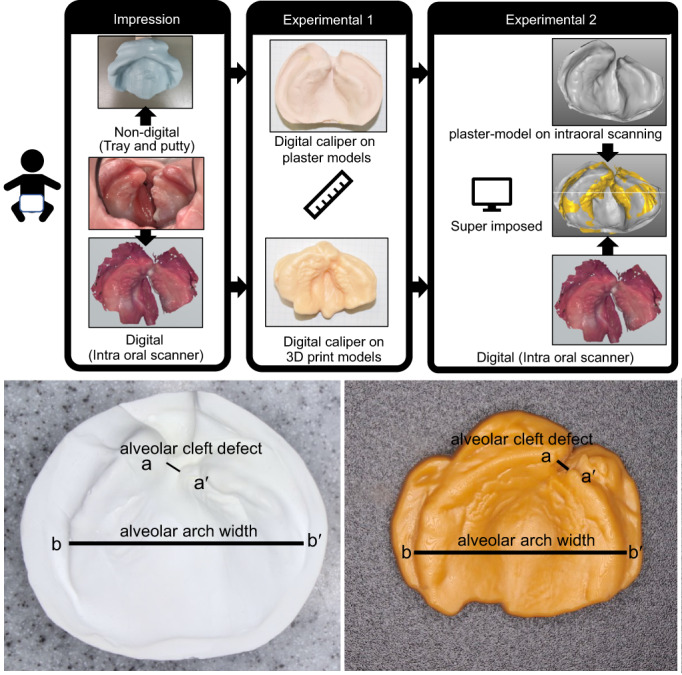
Measurement item; a–a′: alveolar cleft defect/b–b′: alveolar arch width

**FIGURE 2:**
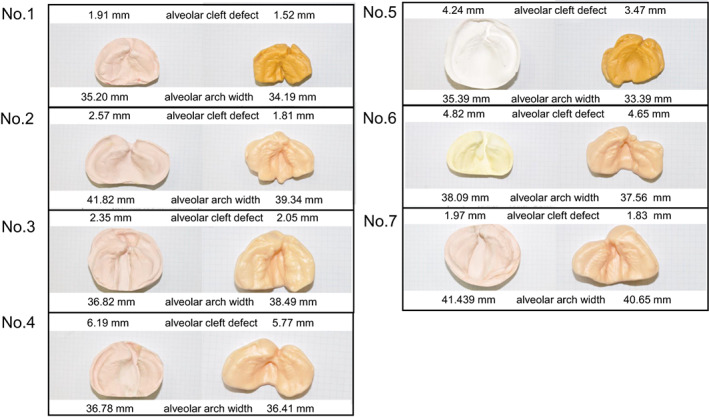
Unilateral cleft lip and palate patient procedure for accuracy verification

Impressions were obtained by the following two steps: (1) Seven patients had palatal impressions obtained using an additive silicone rubber impression material (EXAFINE PUTTY TYPE, GC), and plaster models were used to make a cast model of hard dental stone (NEW PLASTONE II, GC Corporation) or super‐hard dental stone (NEW FUJIROCK IMP, GC Corporation). Cast models were created by mixing the stone powder with water according to the manufacturer's instructions and pouring this mixture into each impression (plaster model group) (for Experiment 1). Plaster model groups were scanned using the D800 (3shape) scanner. Subsequently, the plaster model scan data of the seven patients were then used for standard triangulated language (STL) (for Experiment 2). (2) After 1–2 weeks, the seven patients were directly scanned intraorally using an intraoral 3D scanner (3M True Definition Scanner Trios3, 3Shape Orthodontics Lab R900L). The scan data of the seven patients were then used for STL (for Experiment 2). The intraoral scanner data created an epoxy resin model of each infant's palatal area (dima Print Cast; Kulzer North America) using a high‐resolution 3D printer (cara Print 4.0, Kulzer) (3D printer group) (for Experiment 1) (Figures 1 and 2).

### 2.2 Experiment 1

#### 2.2.1 Measurement method

**FIGURE 3:**
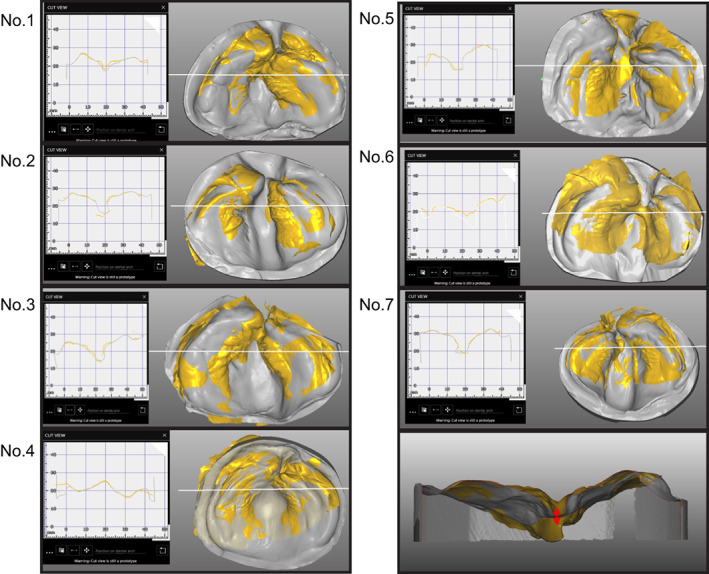
Measurement of alveolar cleft defect and alveolar arch width in the plaster model and 3D printer groups

The plaster and 3D printer models used for NAM were measured directly three times using an electronic digital caliper (Mitutoyo Co.), and the average value was used as data. The measurement site is shown in Figures 1 and 3, and the values are given in Tables 1 and 2. For the width of the cleft palate, the distance between the stumps of both segments was measured, whereas, for the maximum width of the alveolar arch, the maximum width between the left and right alveolar crests was measured.

**TABLE 1:** Mean values, medians, and standard deviations (SDs) of measurements calculated for alveolar cleft defects

Case No.	Cleft type	Sex	Models using dental stones			Intraoral scanner and digital models			M‐W
			Alveolar cleft defects						
			Mean (mm)	Median (IQR)	SD	Mean (mm)	Median (IQR)	SD	
1	LUCLP	Male	1.91	1.91 mm (0.0)	0.0153	1.52	1.52 mm (0.0)	0.0208	N‐S
2	LUCLP	Male	2.57	2.60 mm (0.1)	0.0551	1.81	1.81 mm (0.0)	0.0153	N‐S
3	LUCLP	Male	2.35	2.33 mm (0.0)	0.0404	2.05	2.05 mm (0.0)	0.0404	N‐S
4	LUCLP	Male	6.19	6.19 mm (0.1)	0.0115	5.77	5.74 mm (0.0)	0.0404	N‐S
5	LUCLP	Male	4.24	4.24 mm (0.1)	0.0666	3.47	3.47 mm (0.0)	0.0200	N‐S
6	LUCLP	Female	4.82	4.82 mm (0.0)	0.0153	4.65	4.65 mm (0.0)	0.0153	N‐S
7	RUCLP	Female	1.97	1.97 mm (0.0)	0.0058	1.83	1.82 mm (0.0)	0.0208	N‐S

*Note*: The Mann–Whitney *U* test conducted on the plaster model and 3D printer groups reveals no significant difference in the values of the alveolar cleft defect between the groups.

Abbreviations: IQR, interquartile range; LUCLP, left unilateral cleft lip and palate; M‐W, Mann–Whitney *U* test; N‐S, not significant (Mann–Whitney *U* test; *p* < 0.05); RUCLP, right unilateral cleft lip and palate.

**TABLE 2:** Mean values, medians, and standard deviations (SDs) of measurements calculated for alveolar arch width

Case No.	Cleft type	Sex	Models using dental stones			Intraoral scanner and digital models			M‐W
			Alveolar arch width						
			Mean (mm)	Median (IQR)	SD	Mean (mm)	Median (IQR)	SD	
1	LUCLP	Male	35.20	35.20 mm (0.1)	0.0112	34.19	34.11 mm (0.2)	0.2268	N‐S
2	LUCLP	Male	41.82	41.82 mm (0.0)	0.0153	39.34	39.23 mm (0.2)	0.1935	N‐S
3	LUCLP	Male	36.82	38.61 mm (0.0)	0.0306	38.49	38.55 mm (0.3)	0.3296	N‐S
4	LUCLP	Male	36.78	36.81 mm (0.5)	0.4809	36.41	36.24 mm (0.4)	0.3993	N‐S
5	LUCLP	Female	35.39	35.23 mm (0.2)	0.2801	33.39	33.24 mm (0.3)	0.3407	N‐S
6	LUCLP	Female	38.09	38.14 mm (0.1)	0.1513	37.56	37.53 mm (0.3)	0.3460	N‐S
7	RUCLP	Male	41.39	41.42 mm (0.0)	0.0551	40.65	40.78 mm (0.3)	0.3383	N‐S

*Note*: The Mann–Whitney *U* test conducted on the plaster model and 3D printer groups revealed no significant difference in the alveolar arch width between the groups.

Abbreviations: IQR, interquartile range; LUCLP, left unilateral cleft lip and palate; M‐W, Mann–Whitney *U* test; N‐S, not significant (Mann–Whitney *U* test; *p* < 0.05); RUCLP, right unilateral cleft lip and palate.

### 2.3 Experiment 2

#### 2.3.1 Superimposed testing

**FIGURE 4:**
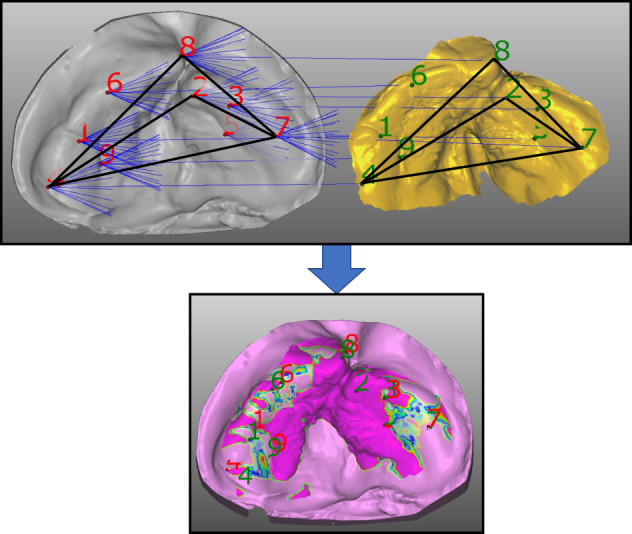
Superimposed cross‐sections of vertical deviations of the plaster model and 3D printer groups

As for the research materials, the plaster and 3D printer models used for NAM were converted into STL data and then superimposed. The two images were superimposed based on two criteria: the alveolar cleft defect and the alveolar arch width, which were the same as those measured in Experiment 1 (Figure 4).

### 2.4 Statistical analyses

We used Levene's test to analyze the collected data for equality of variance by using a statistical software (IBM SPSS Statistics, version 25.0, IBM). When homoscedasticity was not observed, the Mann–Whitney *U* test was performed to analyze the differences between two digital and analog models. The significance level was set at 0.05 for all analyses.

## 3 RESULTS

### 3.1 Experiment 1

#### 3.1.1 Comparative models with dental stone, intraoral scanner, and digital model for each measurement item

Tables 1 and 2 display the medians, standard deviations, and statistical analyses of the collected data. The alveolar cleft defect width ranged from 1.52 to 6.19 mm, and the median alveolar arch width ranged from 33.24 to 41.82 mm. In the first experiment, we used the Kolmogorov–Smirnov test for the measurement values and did not find a normal distribution for both groups (i.e., the alveolar cleft defect and alveolar arch width groups). Subsequently, the Kruskal–Wallis test revealed a significant difference between the alveolar cleft defect and alveolar arch width groups (*p* < 0.05).

The results of the measured values and statistical analyses are summarized in Tables 1 and 2. The Mann–Whitney *U* test conducted on the plaster model and 3D printer groups revealed that comparing the values of the alveolar cleft defect and alveolar arch width groups did not show significant differences between the groups.

### 3.2 Experiment 2

#### 3.2.1 Superimposed testing

The relationship between the superimposed cross‐sections of the digital model (plaster model group) and intraoral scanner (digital group) was evaluated using STL data listed in Figure 4. The superimposed vertical deviations range from 52.9% to 95.7%. In terms of the depth of the alveolar cleft defects, the measurements of the plaster model group (STL) were deeper than those of the digital group. In terms of the width of the alveolar cleft defects, the measurements of the plaster model group showed a wider range than the 3D printer group (Figure 4). The results of the measured values and statistical analyses are summarized in Table 3. The Mann–Whitney *U* test conducted on the plaster model (STL) and intraoral scanner (STL) groups revealed a significant difference in the values of the alveolar cleft defect depth between the groups (*p* < 0.05) (Table 3).

**TABLE 3:** Comparison of superimposed cross‐sections between models using dental stones and intraoral scanner and digital models (STL) the alveolar cleft defect depth

Case no.	Cleft type	Sex	Superimposed area (%)	M‐W
1	LUCLP	Male	58.6	S
2	LUCLP	Male	93.1	NS
3	LUCLP	Male	75.8	S
4	LUCLP	Male	67.1	S
5	LUCLP	Female	95.7	NS
6	LUCLP	Female	52.9	S
7	RUCLP	Male	90.6	NS

*Note*: The abbreviation “S” indicates that the difference between the binary conditions after thermocycling bond strengths is significant (Mann–Whitney *U* test; *p* < 0.05).

Abbreviations: LUCLP, left unilateral cleft lip and palate; M‐W, Mann–Whitney *U* test; N‐S, not significant (Mann–Whitney *U* test; *p* < 0.05); RUCLP, right unilateral cleft lip and palate.

## 4 DISCUSSION

This study evaluated the accuracy verification of different measurement methods (caliper method or on STL data) for conventional and optical impression‐taking using an intraoral scanner and examined whether combining the use of 3D morphometric model and a digital model using an intraoral scanner could prove useful for safe preoperative jaw correction in children with CLP. Moreover, we aimed to compare the efficacy of this new approach to that of the conventional plaster‐mold model. In studies analyzing the maxillary alveolar morphology of patients with CLP, measurement using calipers is common; however, maxillary alveolar morphology measured three‐dimensionally is rarely reported.^6^, ^10^ In this study, a plaster model (plaster model group), for which additional silicone rubber impressions were obtained, and a 3D printer model (3D printer group) were based upon optical impressions obtained for the unilateral lipomaxillary fissure in the same patient. In Experiment 1, the values obtained by the traditional electronic digital calipers showed no significant difference between the plaster model and 3D printer groups (Figure 1). Measurements of the alveolar cleft defect and alveolar arch width, when compared using conventional electronic digital calipers, showed no significant differences between the plaster model and the 3D printer groups. The reason for this is presented in Figure 2. The two measurement sites of alveolar crest defect and alveolar arch width were not significantly different, suggesting that they are not affected by the pressure applied during impression taking. However, in Experiment 2, the relationship between the superimposed cross‐sections of the plaster model (STL) and the intraoral scanner evaluation (STL) was observed to be significantly different in the longitudinal direction, which was statistically significant. The alveolar cleft defect depth was found to be in error between the plaster model (STL) and the intraoral digital model (STL). This factor may be attributed to the pressure application due to the hardness of the putty‐type silicone impression material, as all seven patients showed smaller values for the optical impression compared to those in the plaster models made from the impressions taken using silicone impression material. 3D measurements in this experiment revealed changes in the depth of the alveolar cleft defects that were difficult to identify using the calipers. Moreover, no deformation was observed because the optical impression did not induce pressure. Patel et al. reported that the premaxillary segment showed a degree of variation, which may be explained by the increase in the narrowest part of the cleft palate due to pressure from the silicone rubber impression material. They also suggested that dimensional accuracy in the machining accuracy of the 3D printer model may have been significant.^11^

In this study, four patients had a cleft palate that was corrected to an average of 2.0 mm by preoperative jaw correction, which was almost similar to the treatment goal considered by Grayson et al.^5^, ^6^ After comparing the results of both the conventional and optical impression methods in the same patient, we found that a shift to the 3D printer model is a safe alternative for preoperative jaw correction, as evidenced from the amount of tissue displaced due to the pressure applied during impression taking. In the future, we would like to conduct clinical research with a larger sample size of patients with CLP to further corroborate our current findings.^12^ Weise et al.^13^ reported that impression taking in vulnerable patients can be potentially life‐threatening, with the risk of airway obstruction and aspiration of impression material. The advantages of digital dentistry are being increasingly demonstrated.

In contrast, Burzynski et al. performed three different types of impressions on 180 orthodontic patients. Regarding the impression time, the results showed that the conventional method was slightly faster.^14^ In the present study, the infant required a lot of assistance because of its body movements and inability to keep its mouth open. The wetting of the saliva and the 3D shaking during imaging did not affect the accuracy of the results of this study but significantly prolonged the imaging time.

In conclusion, if this technique can be applied to infants with CLP, it will reduce the risk of vomiting, aspiration, and choking in these patients, thereby reducing the burden on the patients and their guardians. Furthermore, it may have a positive impact on the outcome of preoperative orthognathic treatment. This study validates the accuracy of the conventional method and optical impression taking and contributes to the efficient provision of treatment for children with CLP. Therefore, the strategy of undertaking 3D printer modeling may be beneficial for the treatment of CLP in the future.

## CONFLICT OF INTEREST

The authors declare no conflict of interest.
